# Non-enzymatic Transformation of Aflatoxin B_1_ by *Pseudomonas geniculata* m29

**DOI:** 10.3389/fmicb.2021.724103

**Published:** 2021-08-10

**Authors:** Yuanyuan Yao, Xian Shu, Dongdong Wang, Wenjie Kan, Pengfei Su, Hao Hu, Xu Chen, Dacheng Wang, Shengwei Huang, Lifang Wu

**Affiliations:** ^1^Key Laboratory of High Magnetic Field and Ion Beam Physical Biology, Hefei Institutes of Physical Science, Chinese Academy of Sciences, Hefei, China; ^2^School of Life Sciences, University of Science and Technology of China, Hefei, China

**Keywords:** aflatoxin B_1_, *Pseudomonas geniculata* m29, transformation, non-enzymatic, LC-MS analysis

## Abstract

Aflatoxin B_1_ (AFB_1_) is the most harmful mycotoxin produced by filamentous fungi and presents a serious threat to human and animal health. Therefore, it is essential to protect humans and animals from AFB_1_-induced acute and chronic toxicity. In this study, *Pseudomonas* strain m29 having a high efficiency of AFB_1_ transformation was isolated from soil. The transformation ratio by m29 was more than 97% within 24 h, and the optimum temperature for transformation was 37°C. Moreover, the AFB_1_ transforming activity was mainly attributed to the cell-free supernatant of strain m29. The metabolite that plays a crucial role in AFB_1_ transformation is likely 1,2-dimethylhydrazine or 1,1-dimethylhydrazine, as identified by GC-MS and LC-MS analysis. AFB_1_ was transformed into a product with molecular formula C_17_H_14_O_7_. To the best of our knowledge, this is the first study of non-enzymatic AFB_1_ transformation by bacteria. Importantly, this AFB_1_ transformation mechanism could be universal to various microorganisms.

## Introduction

Aflatoxins are a class of highly toxic secondary metabolites produced mainly by genera of *Aspergillus*, including *Aspergillus flavus*, *Aspergillus parasiticus*, *Aspergillus tamari*, and *Aspergillus nominus*, under both field and storage conditions (prefer to 20–35°C and relative humidity above 89%) (Diener and Davis, 1967; Kurtzman et al., 1987). Aflatoxin B_1_, B_2_, G_1_, and G_2_ are the most common among more than 20 kinds of aflatoxins, of which Aflatoxin B_1_ (AFB_1_) is the most toxic and carcinogenic (Gourama and Bullerman, 1995). Aflatoxin contamination of food and feed results in significant economic losses worldwide and seriously threatens human health. Therefore, considerable interest has been focused on finding effective AFB_1_ detoxification methods for food safety (Strosnider et al., 2006).

Various methods, including several physical and chemical strategies, have been proposed for the removal of aflatoxin contamination to manage the economic losses and health risks caused by the toxin, such as absorption, irradiation, ozone treatment, and sodium bisulfite treatment (Hagler et al., 1983; Diaz et al., 2004; Isman and Biyik, 2009; Kamber et al., 2017). In recent years, researchers have focused on microbial detoxification of aflatoxin due to its unique advantages like minimum loss of product qualities, mild processing conditions, and low cost (Verheecke et al., 2016; Raksha Rao et al., 2017). Over the past decades, several bacterial or fungal strains, such as *Rhodococcus erythropolis* (Alberts et al., 2006), *Bacillus licheniformis* (Wang et al., 2018), *Pseudomonas aeruginosa* (Sangare et al., 2014), *Cellulosimicrobium funkei* (Sun et al., 2015), and *Aspergillus niger* (Zhang et al., 2014), have been developed to remove aflatoxins. However, the industrial application of these strains is limited by some obvious disadvantages, such as low transformation efficiency, narrow operating temperature, and unknown removal mechanism.

In addition, there have been few studies on the mechanism of bacterial transformation of AFB_1_. Almost all studies have shown that bacterial transformation of AFB_1_ is an enzyme-dependent process. The enzymes responsible for AFB_1_ transformation have been identified as oxidase, reductase, and peroxidase (Doyle and Marth, 1979; Wu et al., 2015; Adebo et al., 2017). Aflatoxin oxidase (AFO) was identified in 1998 as the first enzyme known to transform AFB_1_ (Yao et al., 1998). Two F_420_H_2_-dependent reductases (FDR-A and FDR-B) from *Mycobacterium smegmatis* were also reported to catalyze the reduction of the α,β-unsaturated ester moiety of aflatoxins (Taylor et al., 2010). Zhao et al. (2011) found an aflatoxin-transforming enzyme (MADE) from *Myxococcus flavus* ANSM068 that can remove aflatoxin B_1_, G_1_, and M_1_ from a solution. Wang et al. (2011) studied the conversion of AFB_1_ to AFB_1_-8,9-dihydrodiol by manganese peroxidase (MNP) from *Phanerochaete sordida* YK-624, which effectively eliminated the mutagenic activity of AFB_1_. However, no data are currently available on the non-enzymatic AFB_1_ transformation by bacteria.

In this study, *Pseudomonas geniculata* strain m29 was isolated, and the mechanism of AFB1 transformation by strain m29 was explored. This strain transformed AFB_1_ through an extracellular and non-enzymatic reaction, and the metabolite responsible for AFB_1_ transformation was isolated and identified. This study is the first to show the non-enzymatic transformation of AFB_1_ by bacteria.

## Materials and Methods

### Reagents and Medium

Aflatoxin B_1_ was purchased from Sangon Biotech Co., Ltd. (Shanghai, China) and standard solution was diluted with methanol to prepare an AFB_1_ stock solution at 25 ppm. Other reagents were purchased from Sinopharm Chemical Reagent Co., Ltd. (Shanghai, China). Feed was purchased from Baiyi Feed Technology Co., Ltd. (Liuyang, China). Coumarin medium (CM) was prepared according to the method described by Guan et al. (2008). Nutrient broth (NB) medium was used for liquid cultures of bacteria.

### Screening for AFB_1_ Transforming Bacteria

Nine soil samples were collected from several wheat fields in Hefei, Anhui Province, China and screened for strains capable of transforming AFB_1_. The preliminary screening was conducted according to the method described by Guan et al. (2008) and Raksha Rao et al. (2017). Colonies that grew well on coumarin medium were considered to possess the ability to transform AFB_1_.

The AFB_1_ transformation ability of the isolates was determined as follows: 16 isolates were incubated with NB containing 0.5 ppm AFB_1_ at 37°C overnight in a gyrotary shaker incubator (180 rpm), and uninoculated NB processed similarly served as a control. The residual AFB_1_ was extracted and detected using high-performance liquid chromatography (HPLC) according to the methods described by Shu et al. (2018). The limit of detection for AFB_1_ (3σ criterion of blank) is 0.2 ppb.

### Identification of Isolates

The genomic DNA of isolate m29 was extracted using an EasyPure Bacteria Genomic DNA Kit (TransGen Biotech Co., Ltd., Beijing, China), and the 16S rRNA gene fragment was amplified using PCR with universal primers (27F and 1492R) and sequenced. The comparison of the obtained sequence with available 16S rRNA gene sequences in the GenBank database was conducted using BLAST program. Five isolates (m6, m36, m29, xls3, and xls8) were collected and identified. Among the monocultures, isolate m29 was selected because of the growth performance and AFB_1_ degrading activity. Physical and biochemical characterization of isolate m29 was performed according to standard methods (Tindall et al., 2007).

### AFB_1_ Transformation by Isolate m29

A culture of m29 was inoculated at 1% (v/v) into 10 ml NB medium. AFB_1_ was added to the culture to achieve the indicated final concentration (0.5 ppm). Strain m29 was incubated at 20, 24, 28, 32, 37, 40, and 42°C for 24 h to determine the effect of temperature on AFB_1_ transformation. The residual AFB_1_ in the samples was calculated to determine the optimal degradation temperature. Then, strain m29 was cultured in NB containing AFB_1_ at the optimal temperature for 72 h, and samples were taken at 0, 3, 6, 12, 24, 48, and 72 h. The residual AFB_1_ was analyzed according to the method mentioned previously.

### *In vitro* Anti-Aflatoxigenic Effect

The anti-aflatoxigenic effect of strain M29 on *A. flavus* stain 3.6305 (*A. flavus*) purchased from the China General Microbiological Culture Collection Center (CGMCC) (aflatoxin producing capacity 422.54 μg/L in liquid culture medium) was determined according to a previously described method (Shu et al., 2018) with minor modification. Briefly, a 200-g pulverized feed containing 5 ml of *A. flavus* spore suspension (1 × 10^6^ CFU/ml) was inoculated with 15 ml of m29 culture (1 × 10^8^ CFU/ml) at 28°C, and the treatment with NB medium was used as the control. Samples were taken after 15 days to detect AFB_1_ by HPLC. In addition, the feed 15 days after inoculation with *A. flavus* was autoclaved at high temperature for 1 h to completely eliminate *A. flavus*. The obtained AFB_1_-contaminated feed was inoculated with m29 culture at 28°C, and samples were taken 7 days later to detect AFB_1_. The treatment with NB medium was used as the control.

### Determination of the Component That Transforms AFB_1_

Aflatoxin B_1_ transformation by different components of the m29 culture, including the supernatant, cells, and cell lysate, was determined according to a previously described method (Xie et al., 2019). Isolate m29 was inoculated into NB and cultured at 37°C for 24 h. The m29 culture was centrifuged at 8,000 × *g* to obtain the supernatant and cells. To obtain cell lysate, the cells were washed twice with phosphate buffer solution (PBS, 0.02 M, pH 7.2) and then disintegrated using an ultrasonic cell disintegrator (Ningbo Xinzhi Instrument Inc., Ningbo, China) for 30 min. After centrifugation at 10,000 × *g* for 10 min, the supernatant was used as cell lysate. Afterward, AFB_1_ with a final concentration of 0.5 ppm was treated with the cell-free supernatant, cells and cell lysates obtained above, respectively. The mixtures were incubated at 37°C for 24 h. The residual AFB_1_ was analyzed according to the method mentioned previously.

### Effects of Incubation Time, Temperature, and Metal Ions on AFB_1_ Transformation by m29 Supernatant

The effects of incubation time, temperature, and metal ions on AFB_1_ transformation were carried out as described by Raksha Rao et al. (2017) with minor modifications. The supernatant was obtained as described previously and exposed to 0.5 ppm AFB_1_. NB processed similarly served as a control. The mixture was cultured at 37°C for 48 h, and samples were taken at 1, 3, 6, 12, 24, 36, and 48 h. The reaction mixture was incubated at 20, 30, 40, 50, or 60°C for 24 h to study the effect of temperature.

Concentrations of 10 mM Cu^2+^ (CuSO_4_), Zn^2+^ (ZnSO_4_), Mg^2+^ (MgCl_2_), Fe^3+^ (FeCl_3_), or Mn^2+^ (MnCl_2_) were added to the mixture to study the effects of metal ions on AFB_1_ transformation, the supernatant without added metal ions served as a control. The residual AFB_1_ was analyzed according to the method mentioned previously.

### Preliminary Analysis of the Metabolite Responsible for AFB_1_ Transformation

#### Effects of Protease K and SDS on AFB_1_ Transformation by m29 Supernatant

The effects of proteinase K and SDS on the AFB_1_ transformation by the supernatant were studied according to the method described by Guan et al. (2008). The supernatant was treated with a concentration of 1 mg/ml proteinase K and 1% SDS. The residual AFB_1_ was analyzed according to the HPLC method mentioned previously.

### Fractionation of Supernatant by Ultrafiltration

The supernatant was ultra-filtered using a Millipore 8050 ultra-filtration unit according to the method described by Zhou et al. (2012) with small modifications. A volume of 50 ml supernatant was filtered through a 3-kDa NMWL membrane to obtain two fractions: a retentate (volume was adjusted to 50 ml as fraction 1, F1; MW > 3 kDa) and a permeate (MW < 3 kDa). The permeate was further subjected to ultrafiltration through a 1-kDa NMWL membrane to produce a second retentate (volume was adjusted to 50 ml as fraction 2, F2; 1 kDa < MW < 3 kDa) and permeate (fraction 3, F3; MW < 1 kDa). The AFB_1_ removal efficiency of the three fractions was determined using HPLC.

#### Preliminary Separation of the Primary AFB_1_-Transforming Metabolite in F3 Fraction

A 50-ml volume of F3 was thoroughly evaporated in a rotary evaporator with a water aspirator vacuum at a rotation speed of 100 rpm and pressure of 150 mmHg in a water bath held at 45°C (Shanghai Yarong Instrument Inc., Shanghai, China) (Cheng, 2003). Then, the liquid in the collecting flask was removed, and its volume was adjusted to 50 ml (named component 1, C1). The residues in the evaporating flask were re-dissolved in 50 ml of distilled water (named component 2, C2). A 50-ml volume of NB medium was treated similarly (named evap-NB). C1, C2, and evap-NB were incubated with AFB_1_ at 37°C for 24 h (the final concentration of AFB_1_ was adjusted to 0.5 ppm) to study the transformation ability of the different components obtained by evaporation. The evap-NB sample containing AFB_1_ served as the control, and the residual AFB_1_ was detected using HPLC.

### Identification of the AFB_1_-Transforming Metabolite Using GC-MS and LC-MS

C1 was prepared as described previously and analyzed using the headspace technique coupled with gas chromatography–mass spectrometry (GC-MS; Gotor-Vila et al., 2017). C1 was incubated at 65°C, and the compounds in the headspace were trapped for 40 min. The trapped compounds were desorbed into the GC injection port at 150°C for 3 min. The oven temperature was set at 50°C for 5 min and then programmed to rise from 40 to 100°C at 20°C/min. The transfer line was heated to 250°C, as was the ion source. The helium carrier gas was set at a flow rate of 1.2 ml/min. The mass spectrometer was operated in electron impact mode at 70 eV, with a scanning range of 30/300 m/z. Volatile compounds were tentatively identified by comparing the mass spectra and the retention times with the data system library (NIST 11 MS Library). The evap-NB was performed under the same conditions as the control, and all measurements were collected with three replicates.

LC-MS analysis was performed using the AGILENT-1200HPLC/6520QTOFMS (United States) system with a C18 analytical column (Gemini 150 × 2.0 mm, particle size 3 μm; Phenomenex). A linear gradient of 5–95% acetonitrile (MeCN)–H_2_O (v/v, 0.1% formic acid) over 15 min was applied to the column, followed by 95 ml MeCN (v/v, 0.1% formic acid) over 5 min with a flow rate of 0.3 ml/min. Mass spectrometry was performed in positive ion mode.

### Identification of Transformation Product

C1, evap-NB, 0.1, and 1% aqueous solution of hydrazine were prepared and treated with AFB_1_ (20 ppm). The samples were then incubated at 37°C for 24 h. Finally, all samples were directly analyzed using LC-MS without chloroform extraction.

### AFB_1_ Transformation by C1 From Other Strains

C1 from strains m6, m36, xls3, and xls8 were prepared using the same method as that for m29 mentioned previously. Similarly, C1 from different strains were incubated with 20 ppm AFB_1_ for 12 h and the products were detected using LC-MS.

### Statistical Analysis

All analyses were performed in triplicate, with the values expressed as mean ± SD. The data were analyzed further using ANOVA at a 95% confidence level followed by Tukey’s test (SPSS 19.0; IBM, United States); differences were considered significant when *p* < 0.05.

## Results and Discussion

### Isolation and Identification of AFB_1_-Transforming Bacteria

In this study, 16 isolates were found to reduce the concentration of AFB_1_ in NB after a 24-h incubation at 37°C, with different effects (Table 1). Six strains had an AFB_1_ transformation ratio of more than 85%, of which isolate m29 had the highest transformation ratio of 89.86%. Thus, this isolate was chosen for further study.

**TABLE 1:** Aflatoxin B_1_ (AFB_1_) transformation ability of screened 16 isolates.

Isolates	AFB_1_ transformation ratio (%)
m2	75.81 ± 3.66
m3	59.48 ± 5.70
m6	87.92 ± 2.14
m12	75.37 ± 8.57
m13	74.14 ± 2.14
m29	89.86 ± 2.42
m30	65.09 ± 4.71
m31	65.87 ± 3.34
m36	85.24 ± 3.62
dy2	79.05 ± 4.72
y2(2)	70.32 ± 1.43
xls1	67.18 ± 2.84
xls2	87.13 ± 1.69
xls3	86.26 ± 3.46
xls8	79.44 ± 4.07
xls9	86.63 ± 3.77

*Isolates are screened from soil samples using coumarin as the only carbon source.*

*Values are expressed as means ± SD (*n* = 5).*

Physiological and biochemical characterization showed that isolate m29 is a Gram-negative bacterium (Supplementary Table 1). The 16S rRNA gene sequence and phylogenetic evolution analysis showed that the closest relative of strain m29 is *P. geniculata* (99% similarity). The resulting sequence was deposited to the GenBank database under the accession number MZ277329. Similarly, m6, m36, xls3, and xls8 were identified as *Pantoea rodasii*, *Pseudomonas taiwanensis*, *Citrobacter portucalensis*, and *Shigella sonnei*, respectively.

Based on the physiological and biochemical characterization results and 16S rRNA gene sequence analysis, isolate m29 was identified as *P. geniculata* m29. Several kinds of *Pseudomonas* have been reported to transform AFB_1_, such as *P. putida* (Samuel et al., 2014; Singh and Mehta, 2019) and *P. aeruginosa* (Sangare et al., 2014). However, this is the first study to report AFB_1_ transformation by *P. geniculata*.

### AFB_1_ Transformation by *Pseudomonas geniculata* m29

**FIGURE 1:**
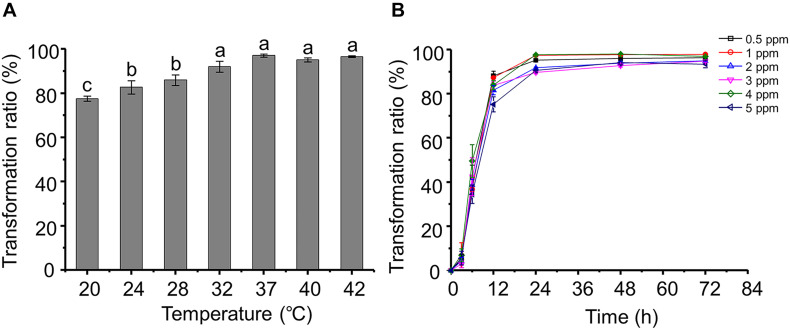
Aflatoxin B_1_ (AFB_1_) transformation characteristics of *Pseudomonas geniculata* m29 (*P. geniculata* m29). **(A)** Effect of temperature on AFB_1_ transformation by *P. geniculata* m29. **(B)** Kinetics of AFB_1_ transformation by *P. geniculata* m29 at 37°C. Values expressed as mean ± SD, and different letters represent significant difference according to Tukey’s LSD test (*p* < 0.05).

Most of the strains that have been reported displayed AFB_1_ transformation activity do so at a narrow temperature range. For example, *A. niger* reduces only 25–45% of AFB_1_ at 20–50°C (Zhang et al., 2014). Interestingly, the AFB_1_ transformation ratios of *P. geniculata* m29 were more than 78% over a wide range of temperatures (20–42°C) (Figure 1A). Moreover, the AFB_1_ transformation ratio reaches a maximum at 37°C, and there was no significant difference between the ratios at 32 and 42°C. A similar result reported by Guan et al. (2008) showed that *S. maltophilia* 35-3 also presented with the highest AFB_1_ transformation ratio at 37°C.

*Pseudomonas geniculata* m29 was incubated with different initial AFB_1_ concentrations (Figure 1B) to study the kinetics of AFB_1_ transformation. The transformation of AFB_1_ by strain m29 was a relatively rapid and continuous process. The transformation ratio of AFB_1_ at 3 h was less than 7%, and AFB_1_ content rapidly decreased from 3 to 24 h. After this period, the concentration of AFB_1_ no longer decreased significantly and remained at a very low level.

The transformation ratio of m29 can reach 97.07% at 24 h, which is the highest rate of microbial transformation in the published literature. For example, *Streptomyces lividans* TK24 can degrade 88% AFB_1_ after 24 h of incubation and *S. aureofaciens* ATCC 10762 by 86% (Eshelli et al., 2015). Harkai et al. (2016) observed a reduction of 88.34% for AFB_1_ by *Streptomyces cacaoi* subsp. *asoensis* after 5 days of incubation. Therefore, strain m29 is a more rapid biocatalyst for AFB_1_ transformation than others reported up to now.

Aflatoxin B_1_ is mutagenic and harmful to bacteria. Significantly, the bacteria capable of AFB_1_ biotransformation can tolerate high doses of AFB_1_. Li et al. (2018) reported that the AFB_1_ transformation ratio of *Candida versatilis* CGMCC 3790 decreased when the initial concentration increased from 10 to 55 ng/g. In contrast, it is clear that the AFB_1_ concentration had no significant effect on the transformation effect of m29, even up to 5 ppm (Figure 1B). Given the high AFB_1_ transformation efficiency and strong tolerance to AFB_1_, m29 might be a potential candidate for AFB_1_ removal in food and feed.

### *In vitro* Anti-Aflatoxigenic Effect

**FIGURE 2:**
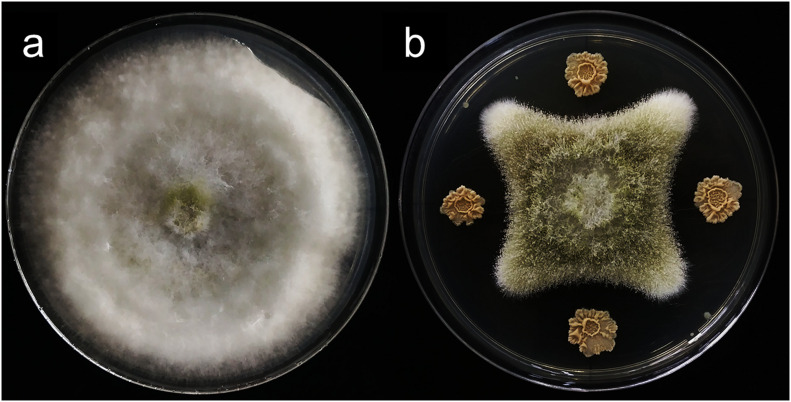
The inhibitory effect of strain m29 on the growth of *Aspergillus flavus* (*A. flavus*). **(A)** *A. flavus* grown for 7 days; **(B)** *A. flavus* was located in the middle of the PDA plate, and m29 was located 3 cm apart away from the center.

*In vitro* antagonistic experiments showed that m29 could significantly inhibit the growth of *A. flavus* (Figure 2). Furthermore, in the feed co-cultured with *A. flavus* and m29, a 75.40% reduction in AFB_1_ can be observed after 15 days (Table 2). In addition, 7 days after inoculating the AFB_1_-containing feed with m29 culture, AFB_1_ decreased by 47.95%. These results further proved that m29 had good application prospects.

**TABLE 2:** Aflatoxin B_1_ (AFB_1_) inhibition in feed containing co-cultures of *Pseudomonas geniculata* m29 and *Aspergillus flavus*.

Treatment groups^*a*^	Conc. of AFB_1_^*b*^ (μ g/g)	AFB_1_ reduction
Feed + NB	0	/
Feed + m29	0	/
Feed + *A. flavus* + NB	2.52 ± 0.42a	/
Feed + *A. flavus* + m29	0.62 ± 0.10b	75.40%
AFB_1_-containing feed^*c*^ + NB	0.73 ± 0.17a	/
AFB_1_-containing feed^*c*^ + m29	0.38 ± 0.09b	47.95%

*Values are expressed as means ± SD (*n* = 5).*

*^*a*^Treatment groups were incubated at 28°C.*

*^*b*^Samples in each treatment were taken after 7 days of incubation, the AFB_1_ content of the sample was analyzed by HPLC.*

*^*c*^The feed 15 days after inoculation with *A. flavus*, then autoclaving at high temperature for 1 h.*

### AFB_1_ Transformation by the Supernatant, Cells, and Cell Lysate

**FIGURE 3:**
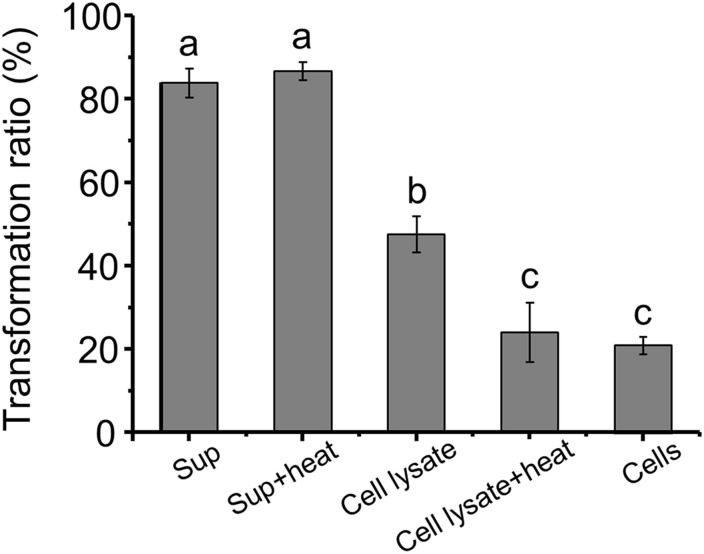
Aflatoxin B_1_ (AFB_1_) transformation by the supernatant, cells and cell lysate of m29 at 37°C after 24 h of incubation. The initial concentration of AFB_1_ was 0.5 ppm. Sup: supernatant; Sup + heat: supernatant treated at 121°C for 20 min; Cell lysate + heat: cell lysate treated at 121°C for 20 min. Values expressed as mean ± SD, and different letters represent significant difference according to Tukey’s LSD test (*p* < 0.05).

The AFB_1_ transformation ratio of the supernatant reached 80% after a 24-h incubation, compared with 46.38 and 20.69% of cells and cell lysate, respectively, (Figure 3), suggesting that the supernatant played a major role in AFB_1_ transformation and the absorption capacity of the cell walls only plays a small role in AFB_1_ removal. It seems that the removal of AFB_1_ by m29 was caused mainly by a metabolite secreted out of the cells, which is in accordance with findings reported for in *Bacillus subtilis* (Xia et al., 2017). AFB_1_ removal by a cell-free supernatant can overcome the disadvantage of using whole cultures that may damage the taste and nutrition of a product (Adebo et al., 2017; Shu et al., 2018). Interestingly, there was no significant change in the AFB_1_ conversion capacity of the supernatant treated at 121°C for 20 min, suggesting that the substances responsible for AFB_1_ in the supernatant may be small molecule compounds or heat-resistant proteins. Similar results were reported by Sangare et al. (2014). In addition, the AFB_1_ transformation ratio of heated cell lysate was significantly reduced, which indicated that intracellular heat-labile components (probably enzymes) also play an important role in AFB_1_ transformation by m29. Similarly, Li et al. (2018) reported that *C. versatilis* CGMCC 3790 transforms AFB_1_ through intracellular heat-labile enzymes.

### Effect of Time, Temperature, and Metal Ions on AFB_1_ Transformation

**FIGURE 4:**
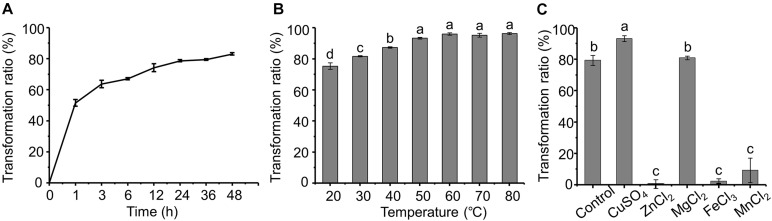
Effect of time, temperature and metal ions on aflatoxin B_1_ (AFB_1_) transformation by supernatant of m29. **(A)** Effect of incubation time; **(B)** effect of temperature; **(C)** effect of metal ions. Values expressed as mean ± SD, and different letters represent significant difference according to Tukey’s LSD test (*p* < 0.05).

The dynamics of AFB_1_ transformation by the cell-free supernatant are shown in Figure 4A. It seems that AFB_1_ transformation by the supernatant of m29 is a relatively rapid process. Most of the transformation occurs within 12 h, and the transformation ratio reached 51.49% after only 1 h of incubation, which is faster than previously reported in other bacteria. For example, Alberts et al. (2006) reported that supernatant of *R. erythropolis* transformed 68.2% of AFB_1_ after 72 h of incubation. Similarly, Song et al. (2019) reported that *P. aeruginosa* M19 removed only 32.8% of AFB_1_ in the initial 6 h, and 80% of AFB_1_ was reduced after 144 h of incubation.

Furthermore, the AFB_1_ transformation ratio of the supernatant increased with temperature, and the transformation ratio reached 93.37% after incubating at 50°C for 24 h (Figure 4B). It is worth noting that even with incubation at 60°C for 24 h, the AFB_1_ transformation ratio by the supernatant was not significantly affected, indicating that m29 transformed AFB_1_ through a heat-resistant enzyme or other metabolite. Similarly, the AFB_1_ transformation ratio of the cell-free supernatant of *Fusarium* sp. WCQ3361 had no significant change at a wide range of temperatures from 0 to 90°C (Wang et al., 2017). The excellent thermal stability means that m29 can stably and efficiently remove AFB_1_ in different applications.

The effect of metal ions on the AFB_1_ transformation ability of the supernatant is shown in Figure 4C. Cu^2+^ can stimulate AFB_1_ transformation, while Zn^2+^, Fe^3+^, and Mn^2+^ notably inhibited the transformation ability of the m29 supernatant. These results indicate that Cu^2+^ may change the structure of the AFB_1_-transforming metabolite in the supernatant and activate its activity. The activation effect of Cu^2+^ and inhibition effect of Zn^2+^ and Fe^3+^ are in agreement with a study of the AFB_1_ transformation ability of the culture supernatant of *B. licheniformis* CFR1 (Raksha Rao et al., 2017).

### Preliminary Analysis of the AFB_1_-Transforming Metabolite

**FIGURE 5:**
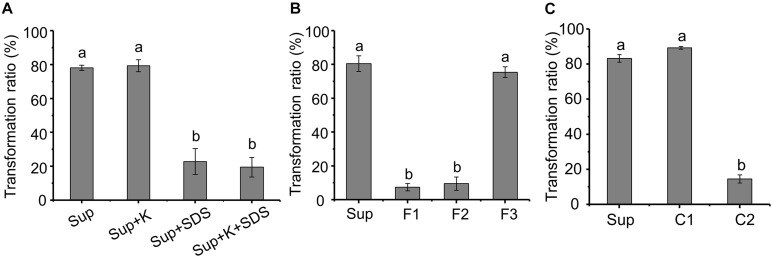
Preliminary analysis of the metabolite transforming aflatoxin B_1_ (AFB_1_). **(A)** Effect of protein K and SDS on AFB_1_ transformation by supernatant. Sup: supernatant; Sup + K: proteinase K treated supernatant; Sup + SDS: SDS treated supernatant; Sup + K + SDS: proteinase K and SDS treated supernatant. **(B)** AFB_1_ transformation by three fractions obtained from supernatant by ultrafiltration. F1, F2, and F3 correspond to samples >3, 1–3, or <1 kDa, respectively. **(C)** AFB_1_ transformation by component obtained from rotary evaporator. Values expressed as mean ± SD, and different letters represent significant difference according to Tukey’s LSD test (*p* < 0.05).

The AFB_1_ transformation ability of the supernatant was not affected by proteinase K, while SDS can significantly reduce the transformation ability (Figure 5A), indicating that AFB_1_ might be transformed by the supernatant of m29 in a non-enzymatic manner. For instance, chemicals such as 1% sodium bisulfite, sodium hydroxide, and aqueous ammonia transform more than 80% of AFB_1_ after 24 h (Moerck et al., 1980).

To identify the metabolite responsible for AFB_1_ transformation, the molecular weight (MW) of the metabolite in the supernatant that transforms AFB_1_ was preliminarily determined using ultrafiltration (Figure 5B). The AFB_1_ transformation ability of F1 and F2 was extremely low, while the AFB_1_ transformation ability of F3 was equal to the untreated supernatant, which indicates that the MW of the AFB_1_-transforming metabolite in the supernatant is lower than 1 kDa. It is very likely that small molecules (less than 1 kDa) or short peptides are responsible for the transformation of AFB_1_.

Components C1 and C2 were obtained from F3 by rotary evaporation, in which the volatile compounds were retained in C1. Figure 5C shows that the AFB_1_ transformation ratio of C1 was 89.25%, while the AFB_1_ transformation ratio of C2 was extremely low (<15%). Furthermore, C1 treated at different temperatures for 1 h were used to transform AFB_1_, and the AFB_1_ transformation ability of C1 treated at 60 and 90°C decreased by 24.12 and 95.34%, respectively, (Supplementary Figure 2). These results suggest that the primary AFB_1_-transforming metabolite produced by m29 is volatile, and AFB_1_ transformation is an extracellular, non-enzymatic reaction. To our knowledge, this is the first report that a volatile compound mediates the microbial transformation of AFB_1_. A similar study conducted by Diniz et al. (2002) reported that the sulfate-reducing bacterium *D. alaskensis* can produce hydrogen sulfide to reduce and decolorize azo dye, which is also an extracellular and non-enzymatic reaction.

### Identification of the AFB_1_-Transforming Metabolite by GC-MS and LC-MS

**FIGURE 6:**
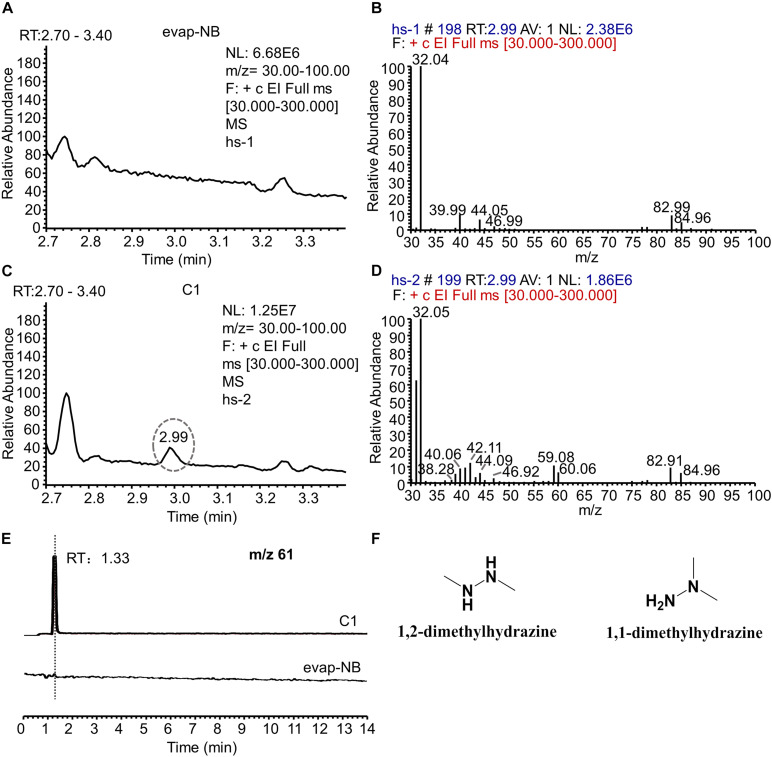
GC-MS and LC-MS analysis of evap-NB and C1. **(A)** Gas chromatogram of evap-NB. **(B)** Mass spectra of peak in chromatogram A at 2.99 min. **(C)** Gas chromatogram of C1; **(D)** mass spectra of peak in chromatogram C at 2.99 min. **(E)** Single ion monitoring of m/z 61. **(F)** Structure of 1,2-dimethylhydrazine and 1,1-dimethylhydrazine. GC-MS, gas chromatography–mass spectrometry; LC-MS, liquid chromatography–mass spectrometry.

Since C1 is the main component with an AFB_1_ transformation ability, the active metabolite in C1 was analyzed using HPLC. However, no new chromatographic peak was observed in C1 at 190–800 nm (Supplementary Figure 3). The headspace coupled with GC-MS was used to analyze the active metabolite with an AFB_1_ transformation ability. The gas chromatogram of evap-NB (control) was shown in Figure 6A and the Figure 6B indicated the mass spectra of peak in Figure 6A at 2.99 min. The peak at 2.99 min (Figure 6C) is a putative AFB_1_-transforming metabolite, and its GC mass spectra are shown in Figure 6D. Comparison with the data system library indicates that the AFB_1_-transforming metabolites might be hydrazine compounds, such as 1,2-dimethylhydrazine and 1,1-dimethylhydrazine. Furthermore, the signal at m/z 61 can only be detected in C1 by LC-MS (Figure 6E), and the HR-ESIMS spectra of this compound are shown in Supplementary Figure 4, which confirmed that the MW of this compound was 60 g/mol. Here, due to the lack of standards for 1,2-dimethylhydrazine and 1,1-dimethylhydrazine, a 0.1% aqueous solution of hydrazine was used to transform AFB_1_. As shown in Supplementary Figure 5, the AFB_1_ transformation product of C1 was the same as that of a 0.1% aqueous solution of hydrazine. In conclusion, these results suggest that the AFB_1_-transforming metabolite of m29 is likely to be 1,2-dimethylhydrazine or 1,1-dimethylhydrazine (Figure 6F). In addition to m29, a variety of microorganisms have been reported to produce various hydrazine-containing compounds, such as katorazone from *Streptomyces* sp. IFM 11299, gyromitrins from *Gyromitra esculenta*, and spinamycin from *Streptomyces albospinus* (Le Goff and Ouazzani, 2014).

### Identification of AFB_1_ Transformation Product

After co-incubation with m29 culture and AFB_1_, chloroform was used to extract AFB_1_ and the transformation product; the structure of the product was further determined by LC-MS (Supplementary Figure 6). However, no transformation product was found, and similar results have been reported by other researchers (Farzaneh et al., 2012; Sangare et al., 2014; Raksha Rao et al., 2017; Xia et al., 2017; Shu et al., 2018). It speculated that the chemical properties of AFB_1_ transformation products are different from those of AFB_1_, making them difficult to be detected (Alberts et al., 2006).

**FIGURE 7:**
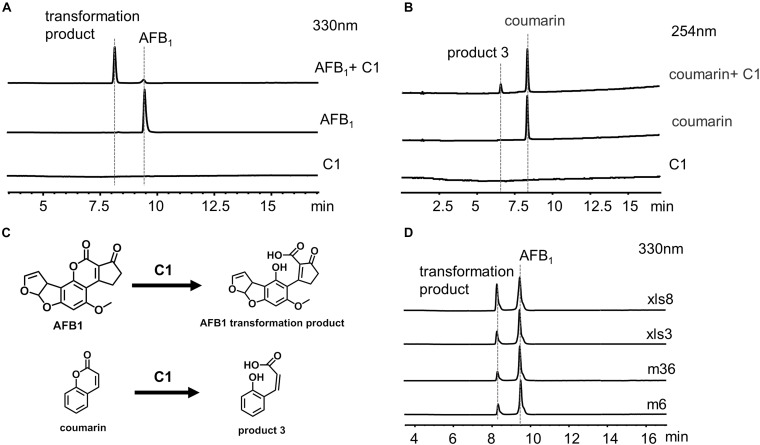
C1 reaction with aflatoxin B_1_ (AFB_1_) and coumarin. **(A)** HPLC analysis of the reaction between C1 and AFB_1_. **(B)** HPLC analysis of the reaction between C1 and coumarin. **(C)** Speculative reaction mechanism of C1. **(D)** AFB1 was transformed by C1 from different strains. AFB_1_ was transformed by C1 from four different strains into the same transformation product as m29. HPLC, high-performance liquid chromatography.

C1 was incubated with 20 ppm AFB_1_ for 24 h, and the transformation product was directly analyzed by LC-MS without extraction using chloroform (Figure 7A) to investigate further the identity of the transformation product. A transformation product with a MW of 330 g/mol (18 units more that of AFB_1_) was observed. The UV and MS data of AFB_1_ and the AFB_1_ transformation product are shown in Supplementary Figures 7, 8. To further determine the structure of the AFB_1_ transformation product, coumarin was reacted with C1, and the product 3 with a MW of 164 g/mol (18 units more that of coumarin) was observed (Figure 7B and Supplementary Figures 9,10). Therefore, the lactone rings of AFB_1_ and coumarin react with C1, rather than the carbonyl group on the five-membered ring of AFB_1_. The structure of transformation product and speculative reaction mechanism is shown in Figure 7C. The same transformation product with unclarified mechanism has also been reported, which was less toxic than AFB_1_ (Qiu et al., 2021). Interestingly, 1% hydrazine could convert AFB_1_ into product two with a MW of 326 g/mol (Supplementary Figure 11), which was speculated to be the product of the reaction of the carbonyl group of AFB_1_ with hydrazine. The UV spectra, MS spectra, and speculative structure of product two are shown in Supplementary Figure 11. The findings also imply that the 1,2-dimethylhydrazine or 1,1-dimethylhydrazine content in C1 might be very low, resulting in the absence of product two in the reaction between AFB_1_ and C1.

### AFB_1_ Transformation by C1 From Other Strains

We hypothesized that the AFB_1_ transformation mechanism might be widespread in a variety of bacteria. Therefore, C1 from four different strains (m6, m36, xls3, and xls8) were used to transform AFB_1_ (Figure 7D). AFB_1_ was transformed into the same product produced by m29 by C1 of four different strains. These results suggest that the transformation of AFB_1_ by C1 may be the first step in a general AFB_1_ detoxification strategy for bacteria. The subsequent AFB_1_ transformation process may require the further involvement of intracellular enzymes.

## Conclusion

In summary, *Pseudomonas* strain m29 that can efficiently transform AFB_1_ was isolated. GC-MS and LC-MS analysis indicate that the transformation process is extracellular and non-enzymatic and mainly depends on the hydrazine compound produced during the growth of bacteria. This is the first study to report the non-enzymatic AFB_1_ transformation by bacteria. In addition, the structure of the AFB_1_ transformation product was preliminarily identified. It is worth noting that the transformation mechanism of AFB_1_ may be widespread in a variety of bacteria, indicating that we should also pay attention to the important role of microbial non-enzymatic transformation in the treatment of aflatoxin contamination. Indeed, future studies are needed to elucidate the AFB_1_ transformation mechanism, and explore the possible use of the *P. geniculata* m29 in food and feed.

## Data Availability Statement

The original contributions presented in the study are included in the article/Supplementary Material, further inquiries can be directed to the corresponding authors.

## Author Contributions

YY, XS, and SH thoroughly discussed and designed this study. YY and XS were in charge of the investigation, data collection, and article writing. DoW analyzed the biological samples. WK, PS, XC, and DaW helped with data curation and formal analysis. LW provided funding and managed the project. All authors contributed to the article and approved the submitted version.

## Conflict of Interest

The authors declare that the research was conducted in the absence of any commercial or financial relationships that could be construed as a potential conflict of interest.

## Publisher’s Note

All claims expressed in this article are solely those of the authors and do not necessarily represent those of their affiliated organizations, or those of the publisher, the editors and the reviewers. Any product that may be evaluated in this article, or claim that may be made by its manufacturer, is not guaranteed or endorsed by the publisher.
