# Supplementary material for: Non-enzymatic Transformation of Aflatoxin B1 by Pseudomonas geniculata m29
Source: Front Microbiol. 2021 Aug 10;12:724103. doi: 10.3389/fmicb.2021.724103 (PMC8383447; doi:10.3389/fmicb.2021.724103)
Supplement: Supplementary file 1 [file Data_Sheet_1.docx]

**Supplementary Information**

Table S1. Physiological and biochemical characteristics of isolate m29

| Characteristic | Result | Characteristic | | Result |
| --- | --- | --- | --- | --- |
| gram stain | - | assimilation of | |  |
| motility | + | glucose | | + |
| catalase | + | maltose | | - |
| oxidase | + | D-Xylose | | + |
| urease | + | galactose | | + |
| acid phosphatase | + | D-Sorbitol | | - |
| nitrate reduction | - | D-Raffinose | | - |
| indole test | - | sucrose | | - |
| V-P test | - | glycerol | | - |
| growth at 5°C | - | lactose | | - |
| growth at 40°C | + | D-fructose | | - |
| growth in 5% NaCl | + | mannitol | | - |
| growth in 10% NaCl | + |  |  | |


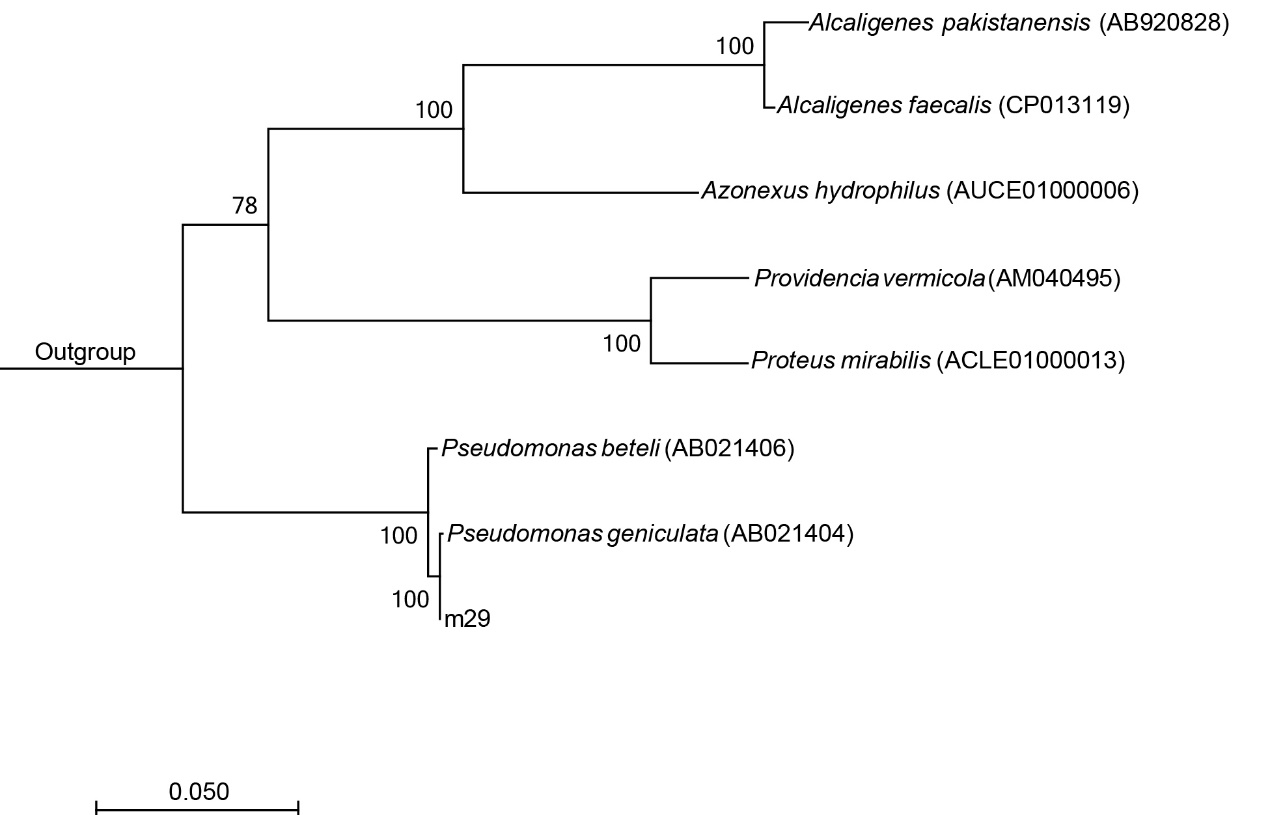


Fig. S1. Phylogenetic tree of isolate m29 based on 16S rRNA gene sequence. *Sphingobacterium zeae* KU201960 was used as an outer group.


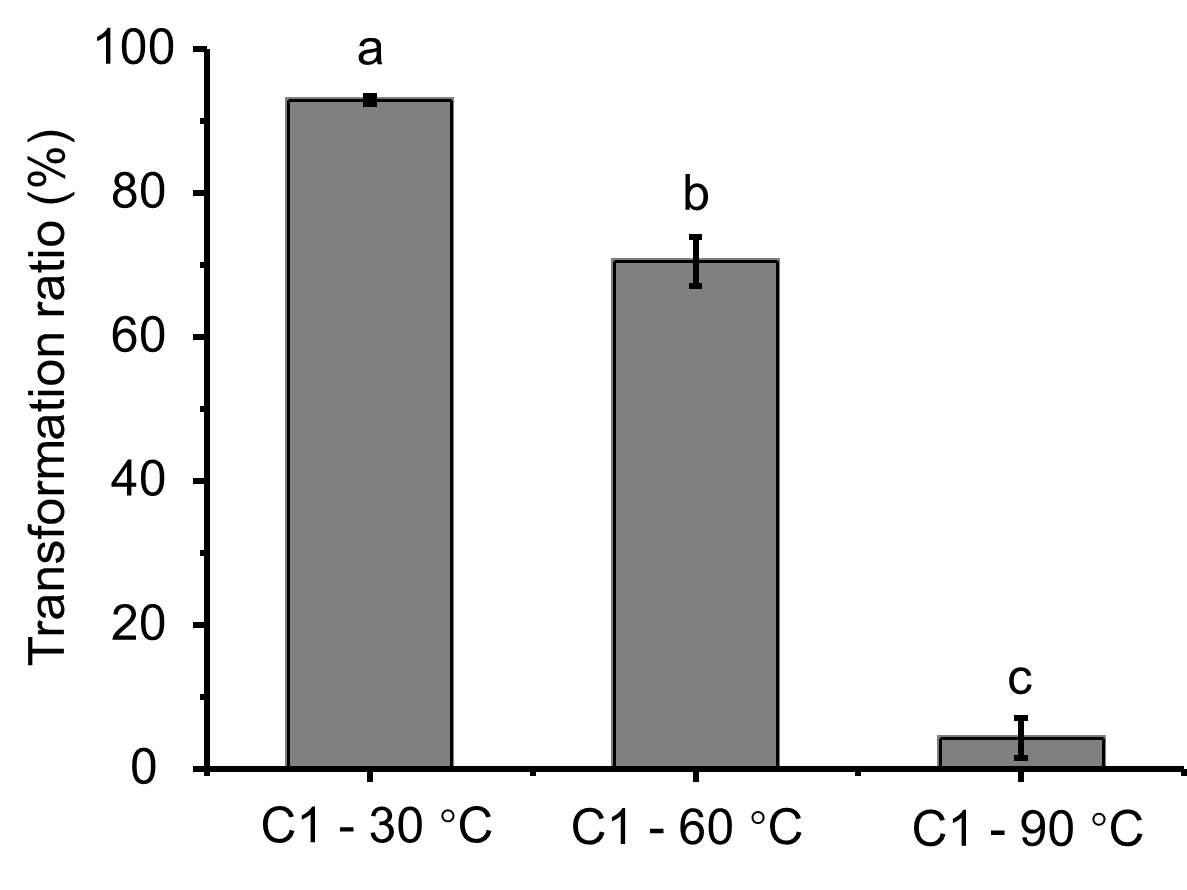


Fig. S2. AFB_1_ transformation ability of C1 treated at different temperatures. C1 - 30 °C: C1 treated at 30 °C for 1 h; C1 - 60 °C: C1 treated at 60 °C for 1 h; C1 - 90 °C: C1 treated at 90 °C for 1 h.


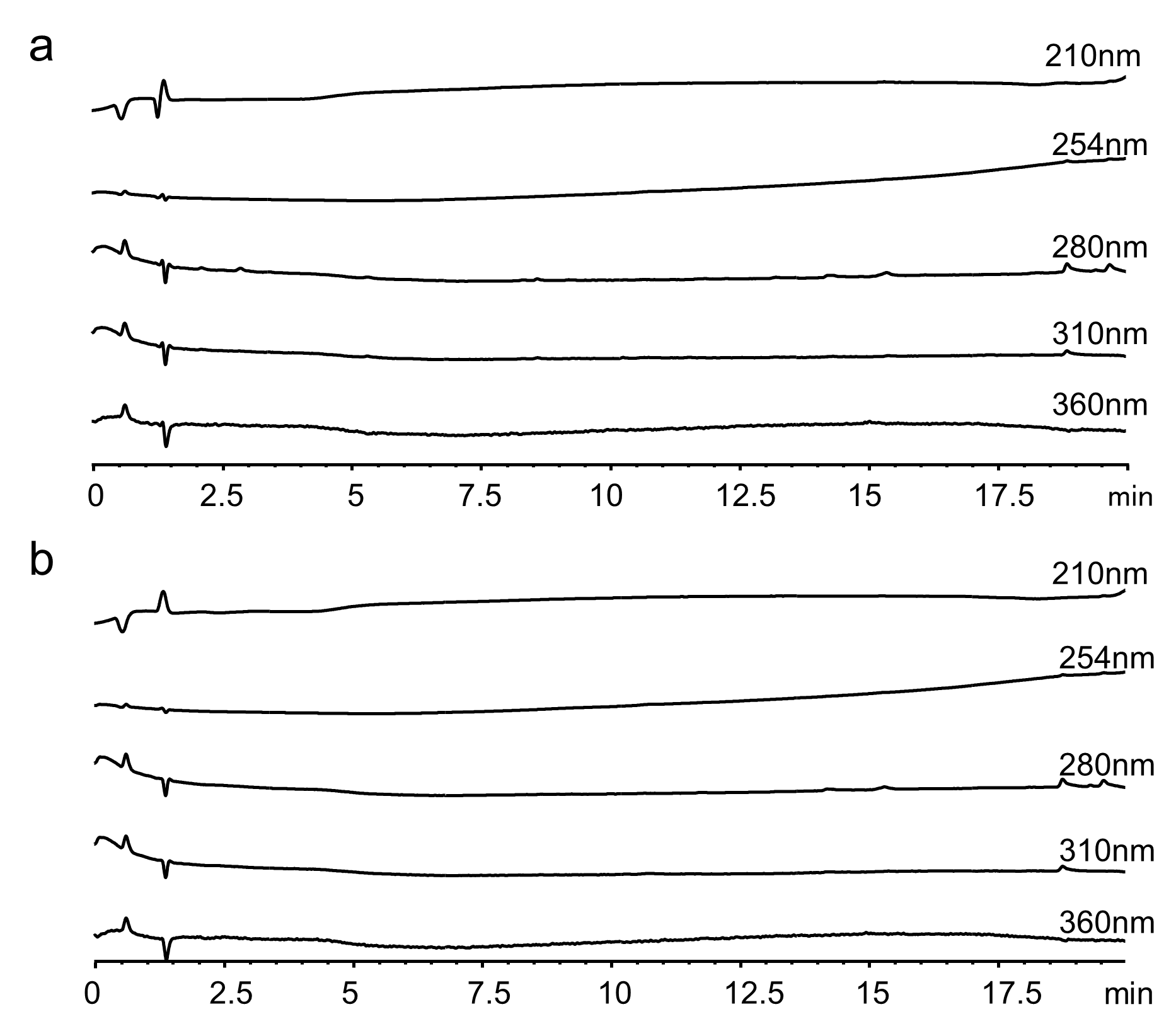


Fig. S3. Chromatograms of evap-NB and C1 at different wavelengths. (a) Chromatograms of evap-NB at 210nm, 254nm, 280nm, 310nm and 360nm; (b) Chromatograms of C1 at 210nm, 254nm, 280nm, 310nm and 360nm. The gradient elution was listed as below: 0-14 min, 10%-100% acetonitrile; 15-18min, 100%-10% acetonitrile and 18-21 min, 10% acetonitrile.


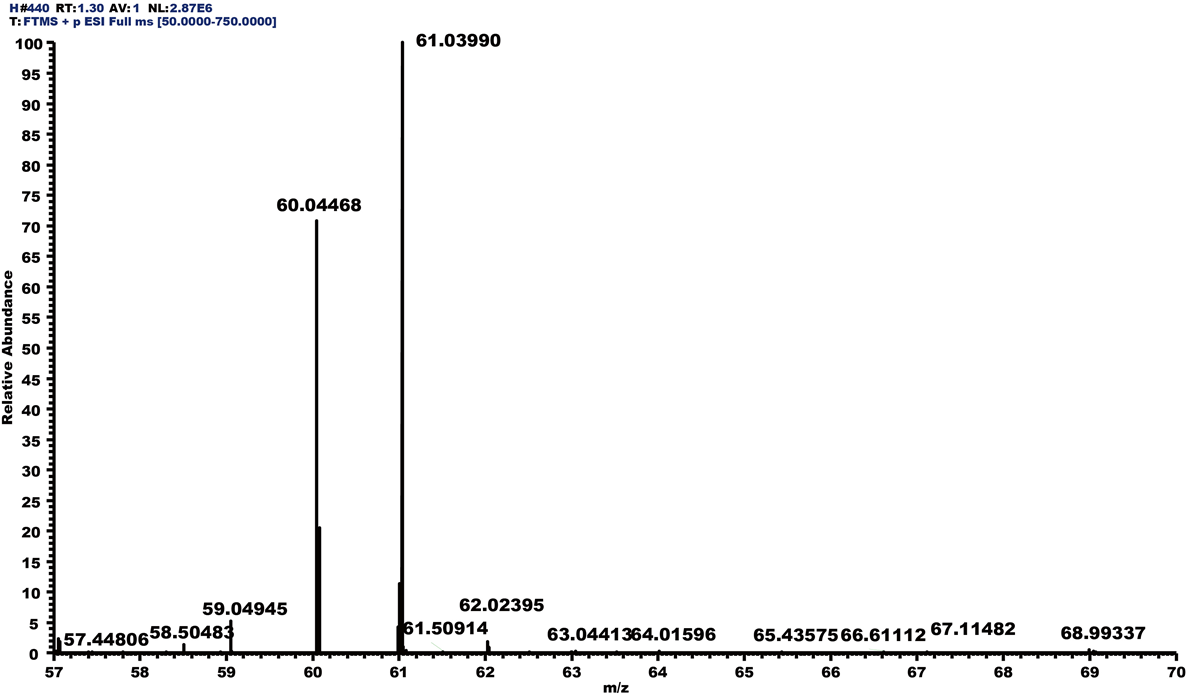


Fig. S4. HR-ESIMS spectra of the substance transforming AFB_1_.


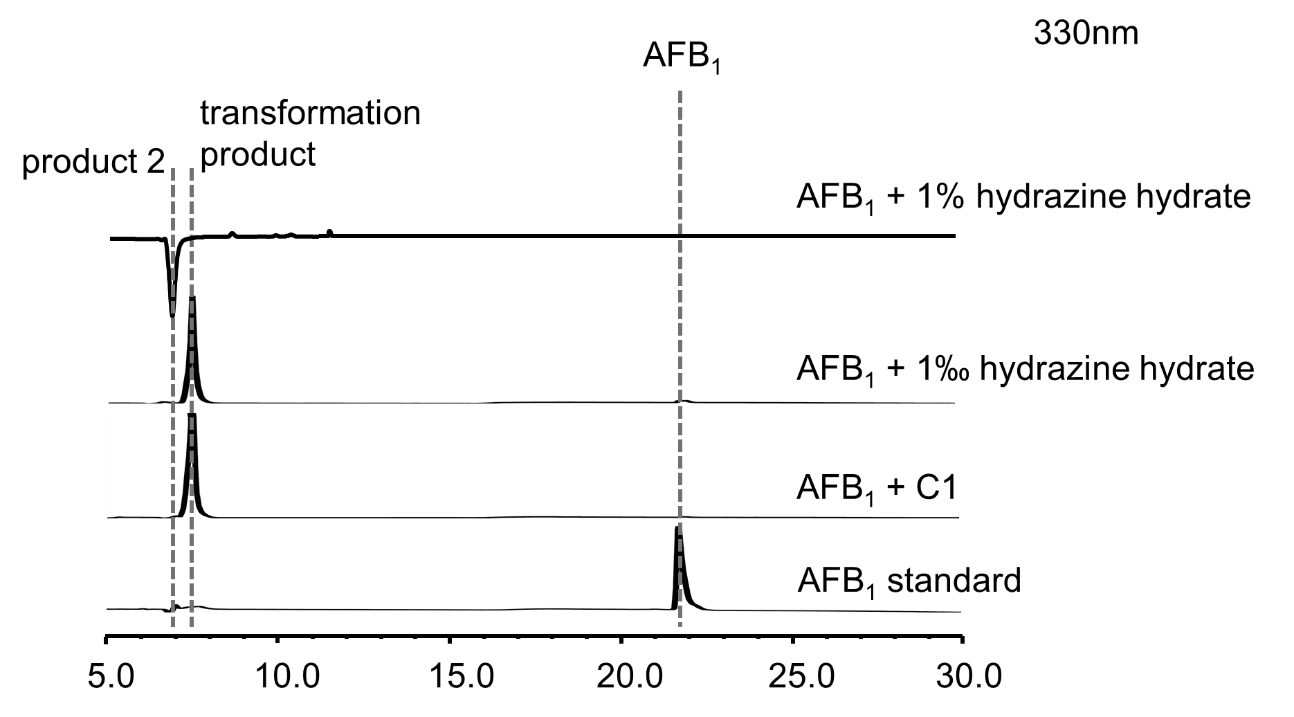


Fig. S5. LC-MS analysis of AFB_1_ transformation product of C1 and aqueous solution of hydrazine. AFB_1_ + C1: 20 ppm AFB_1_ treated with C1; AFB_1_ + 1‰ hydrazine hydrate: 120 ppm AFB_1_ treated with 1 ‰ aqueous solution of hydrazine; AFB_1_ + 1% hydrazine hydrate: 120 ppm AFB_1_ treated with 1 % aqueous solution of hydrazine.


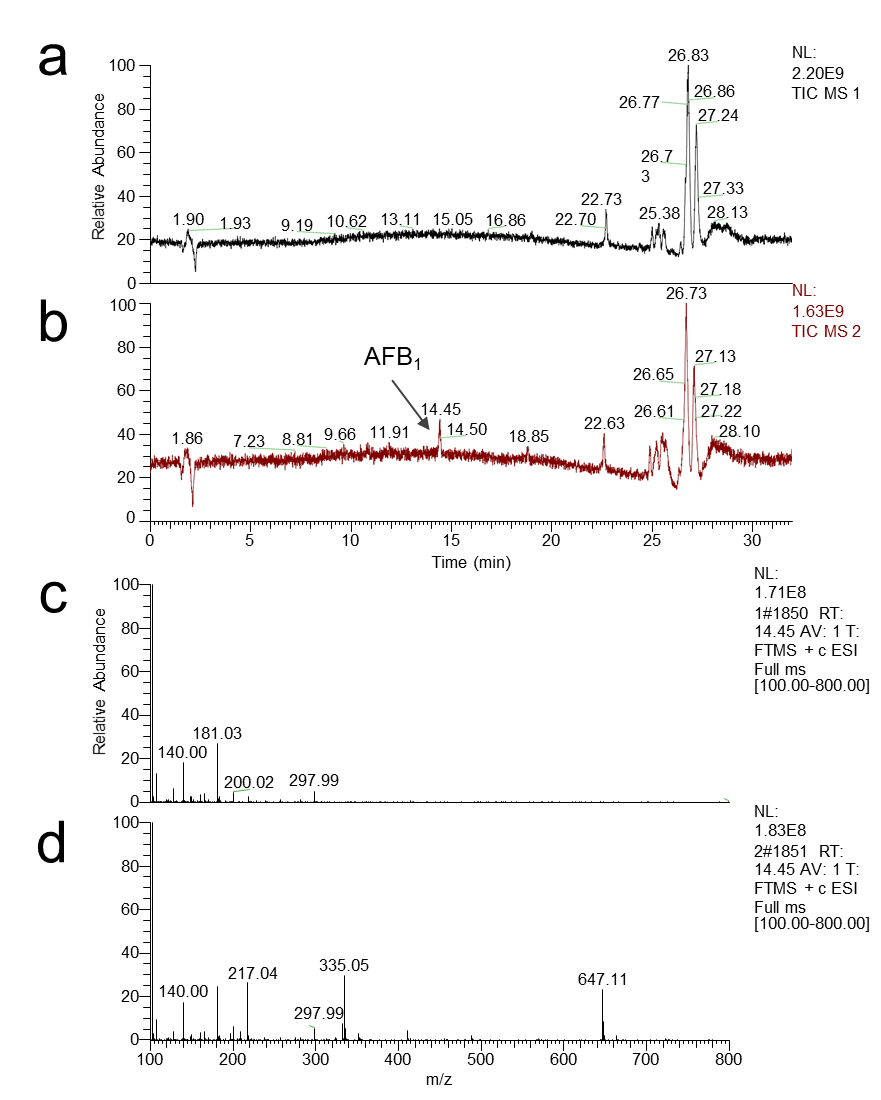


Fig. S6. Mass spectrometric analysis of AFB_1_ transformation by m29 culture. (A) Chromatogram of m29 culture. (B) Chromatogram of m29 culture added AFB_1_. (C) Mass spectrum at 14.45 min in chromatogram A. (D) Mass spectrum at 14.45 min in chromatogram B (AFB_1_).


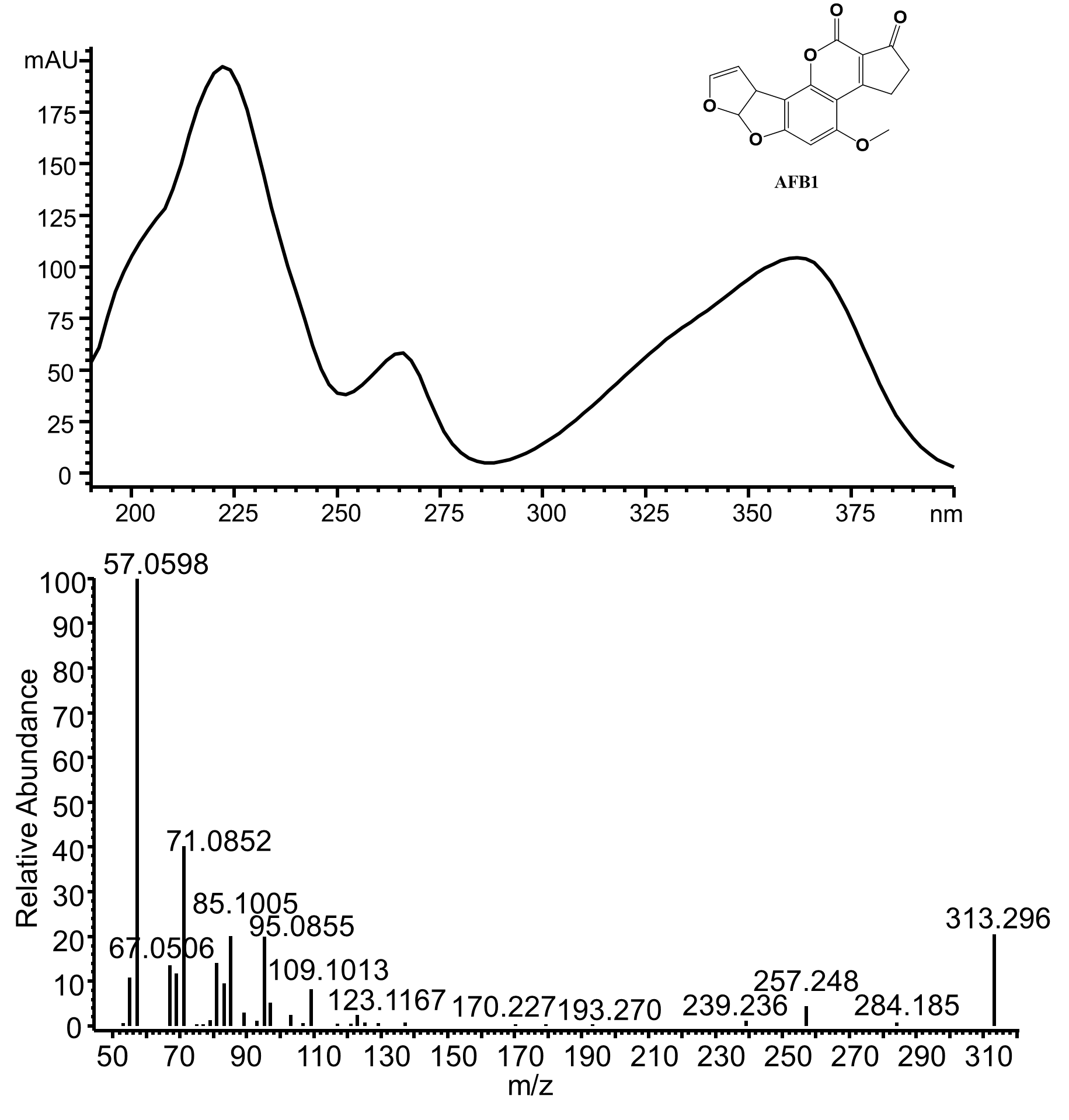


Fig. S7. UV and HR-ESIMS spectra of AFB_1_.


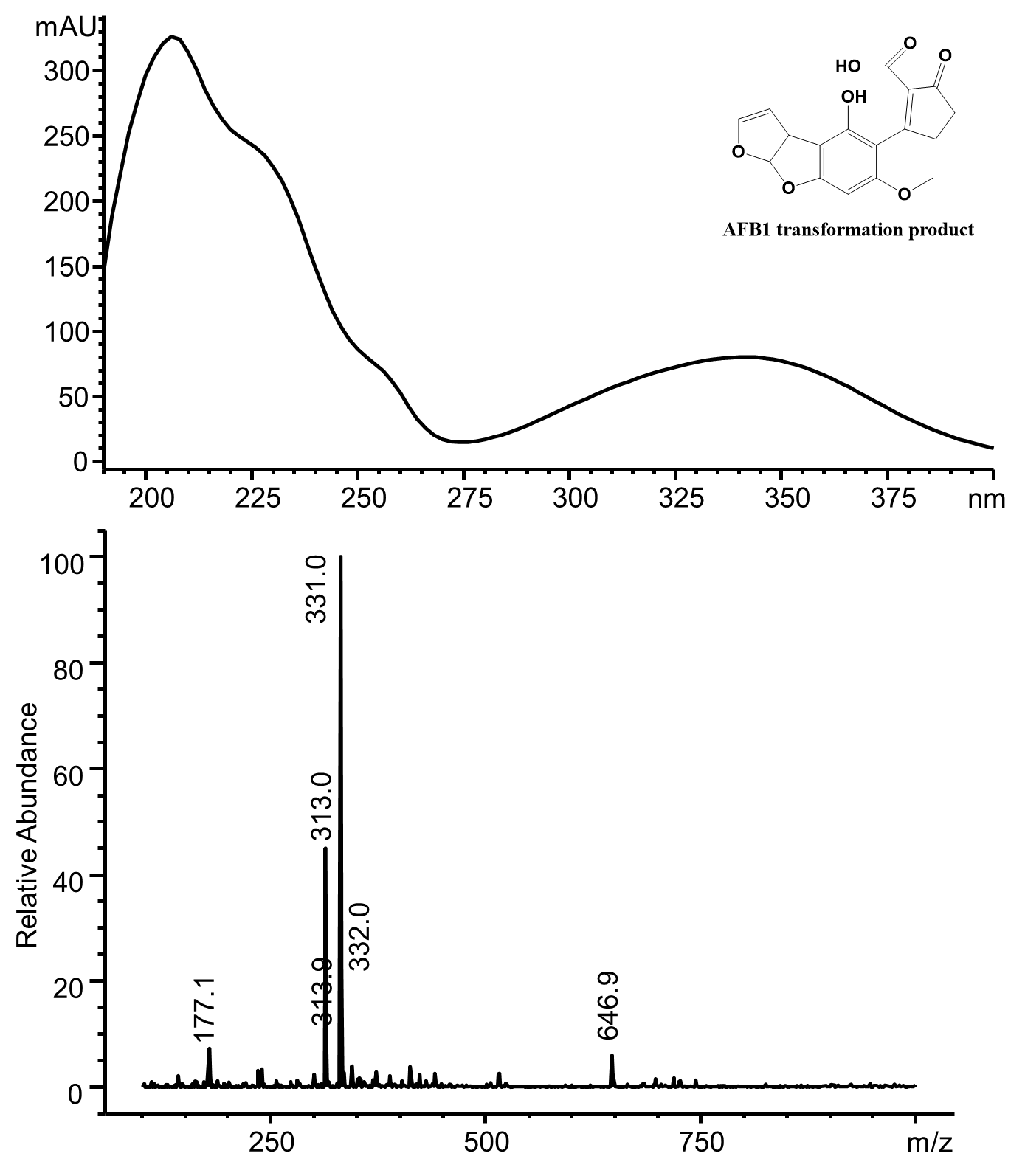


Fig. S8. UV and MS spectra of AFB_1_ transformation product.


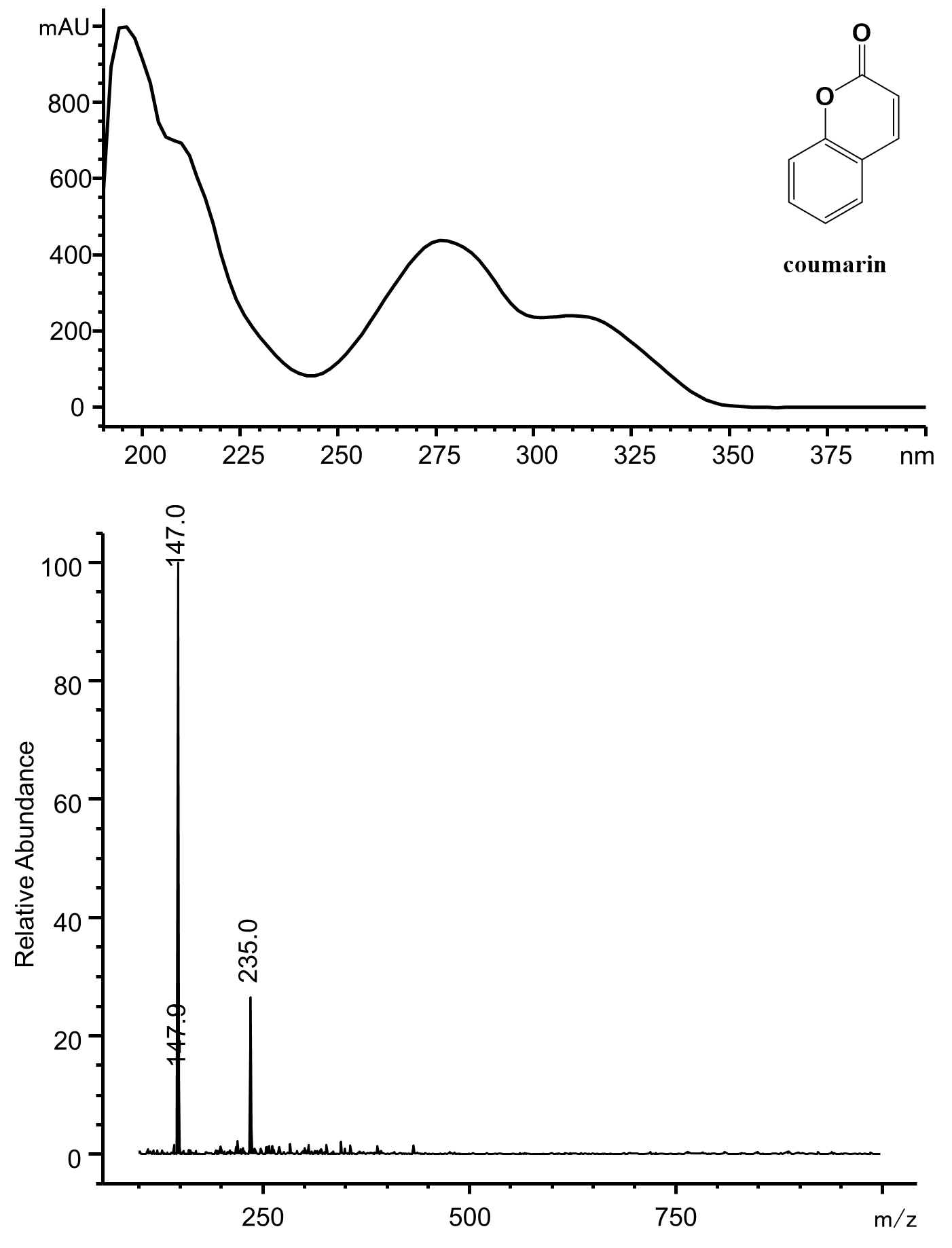


Fig. S9. UV and MS spectra of coumarin.


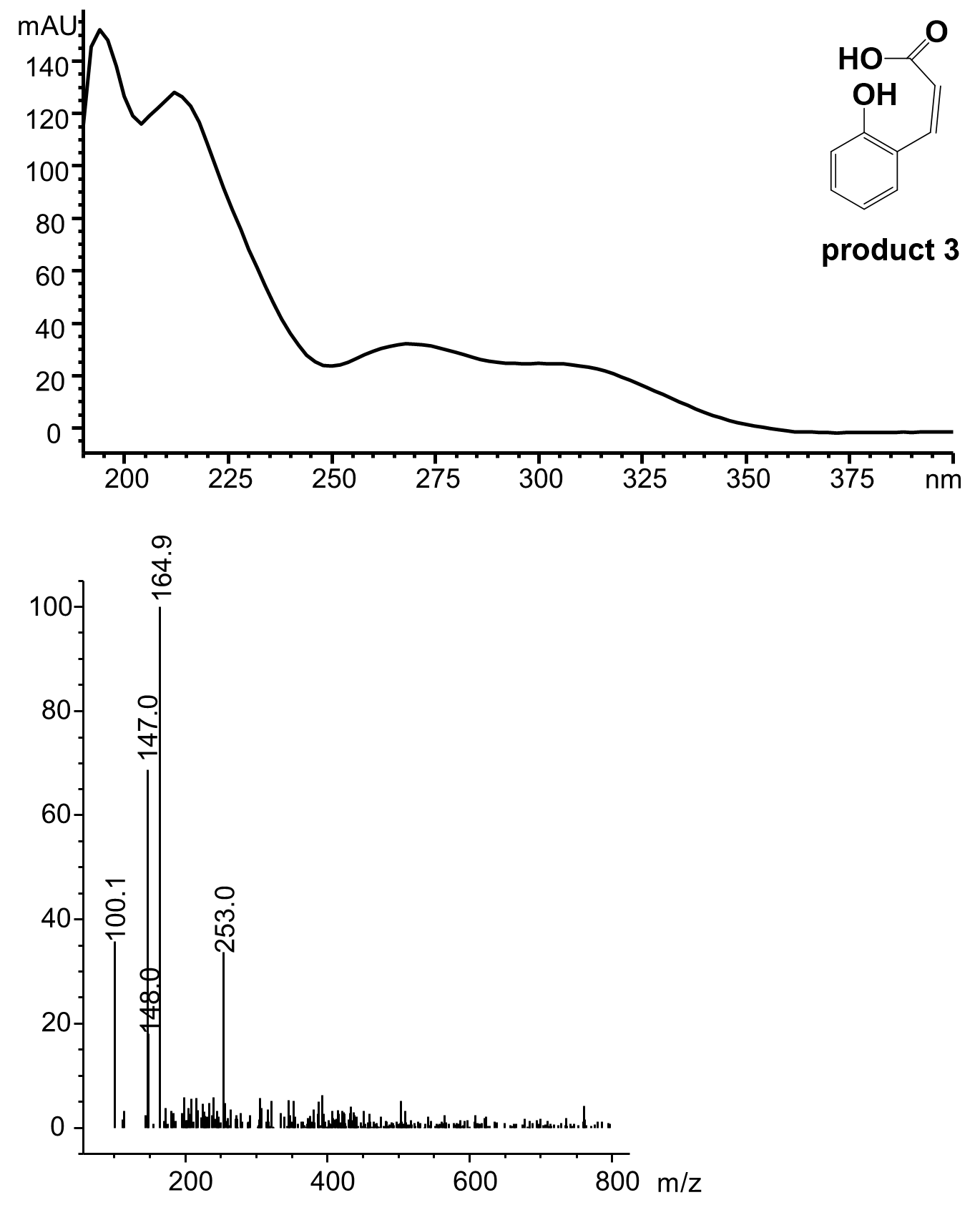


Fig. S10. UV and MS spectra of product 3.


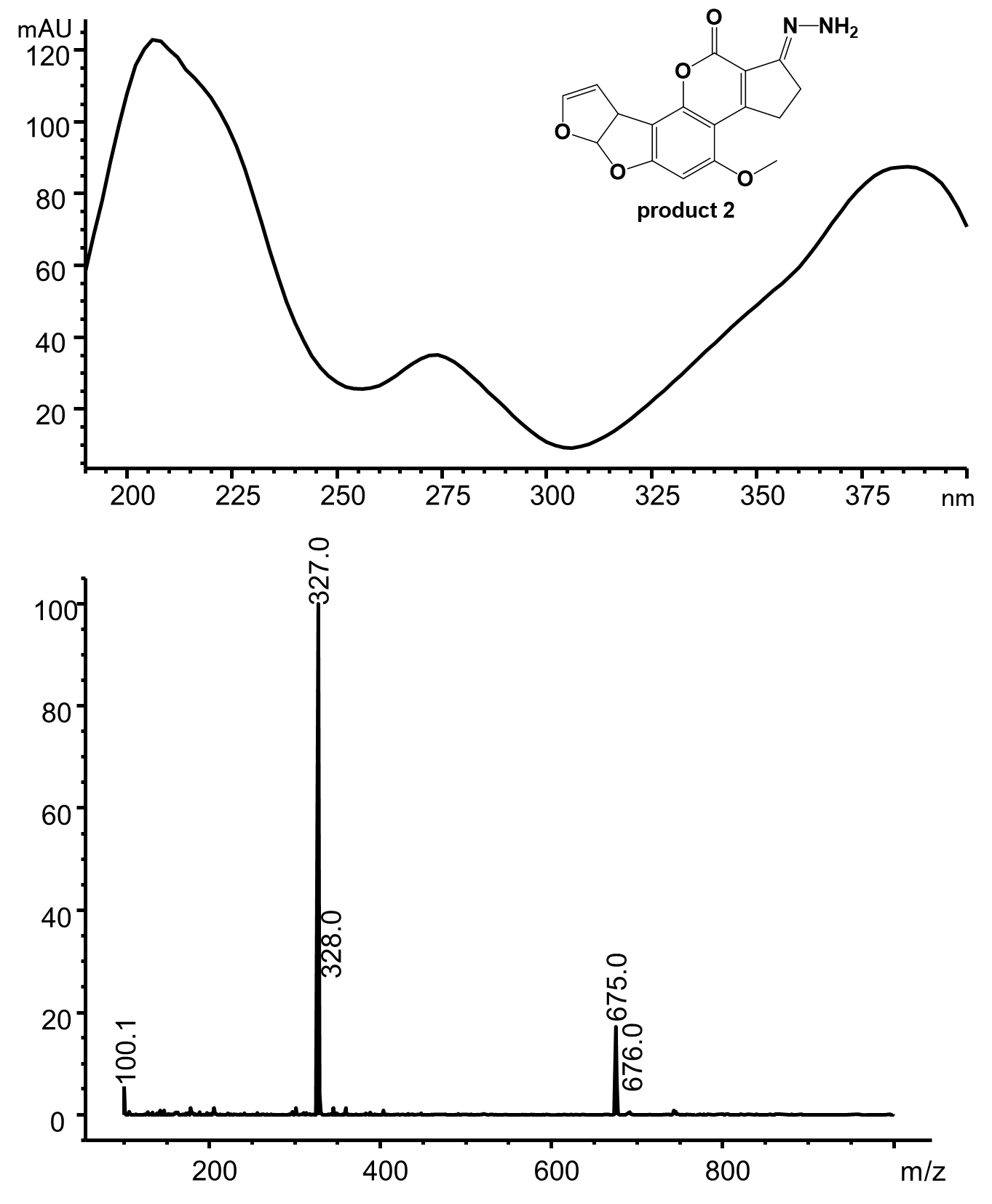


Fig. S11. UV and MS spectra of product 2.


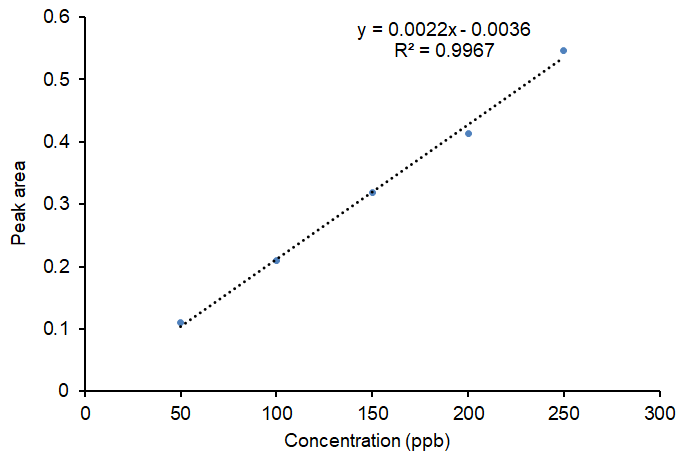


Fig. S12. Standard curve of AFB_1_.
